# Clinical and Radiographic Outcomes of Nitinol Compression Staple Fixation for Midfoot and Chopart Arthrodeses: A Single-Centre Case Series

**DOI:** 10.7759/cureus.110781

**Published:** 2026-06-13

**Authors:** Laraib Zafar, Zain Habib, Ishak Rouf, Ayesha Malik, Oliver Bagshaw, Gary M Hannant

**Affiliations:** 1 Trauma and Orthopaedics, Bradford Royal Infirmary, Bradford, GBR; 2 Trauma and Orthopaedics, Manchester University NHS Foundation Trust, Manchester, GBR; 3 Emergency Medicine, Manchester Royal Infirmary, Manchester, GBR; 4 Trauma and Orthopaedics, Bradford Teaching Hospitals NHS Foundation Trust, Bradford, GBR

**Keywords:** chopart arthrodesis, foot and ankle surgery, internal fixation, midfoot arthrodesis, nitinol compression staples

## Abstract

Introduction: Nitinol continuous compression staples are increasingly used for foot and ankle arthrodesis; however, real-world outcome data describing construct strategies, regional variation in union, and failure patterns remain limited, particularly for Chopart joints.

Methods: A retrospective case series of consecutive adult patients undergoing midfoot or Chopart arthrodesis using nitinol compression staples as the principal fixation method was performed. Arthrodeses were classified as midfoot (tarsometatarsal and/or naviculocuneiform) or Chopart (talonavicular and/or calcaneocuboid). Fixation constructs were categorised as staple-only or hybrid (staples combined with screws and/or plates). Radiographic union was defined as ≥50% trabecular bridging on two orthogonal radiographs or confirmation on computed tomography. The primary outcome was union rate; secondary outcomes included time to union, complications, reoperation, construct configuration, and failure patterns.

Results: Twenty-five arthrodeses were included (15 midfoot and 10 Chopart). Overall union was achieved in 22 of 25 procedures (88%). Union rates were higher in the midfoot cohort (14/15, 93.3%) than in the Chopart cohort (8/10, 80%). Median time to union among united procedures was 62 days (interquartile range: 47-80). Three non-unions (12%) and three complications (12%) occurred, with two procedures requiring further surgery. Staple-only constructs accounted for 20 procedures, with five hybrid constructs (four Chopart and one midfoot). One case of staple fatigue failure occurred at the bridge-leg interface.

Conclusion: Nitinol compression staples provided reliable fixation for midfoot arthrodesis and acceptable outcomes in selected Chopart arthrodesis. Region-specific differences in union and failure patterns highlight the importance of construct planning and selective adjunct fixation for higher-risk Chopart constructs.

## Introduction

Arthrodesis of the midfoot and Chopart joints is an established treatment for painful arthritis, deformity, and instability. Despite advances in fixation techniques, achieving reliable union remains challenging due to complex joint anatomy, variable bone quality, and exposure to multiplanar shear forces, particularly at the talonavicular and calcaneocuboid joints [1].

Nitinol continuous compression staples have gained popularity as a low-profile fixation option that provides sustained dynamic compression through shape-memory recovery. Clinical outcome data have expanded in recent years. Dock et al. reported favourable outcomes for tarsometatarsal arthrodesis using nitinol staples, demonstrating union rates comparable with traditional fixation methods [2]. Horner et al. evaluated isolated nitinol staple fixation across midfoot and Chopart joints and demonstrated a clear regional difference, with higher union rates in midfoot joints compared with Chopart joints [3]. Recent reviews support the efficacy of nitinol staples while highlighting heterogeneity in construct configuration and reporting standards [4]. They have also demonstrated favourable outcomes in forefoot arthrodesis, such as first metatarsophalangeal joint fusion, supporting the broader clinical utility of sustained dynamic compression across the foot [5].

Despite increasing adoption, limited real-world data exist describing outcomes across mixed midfoot and Chopart cohorts outside specialist centres [4]. The purpose of this retrospective single-centre case series was to evaluate radiographic union following nitinol compression staple fixation for midfoot and Chopart arthrodeses. Secondary outcomes included time to union, complications, reoperations, and fixation construct characteristics.

## Materials and methods

A retrospective case series of consecutive adult patients undergoing midfoot or Chopart arthrodesis using nitinol compression staples as the principal fixation method was performed at Bradford Royal Infirmary, a single tertiary centre in Bradford, United Kingdom, between December 2023 and July 2025.

The project was registered locally as a service evaluation and did not require formal research ethics committee approval. Written informed consent was obtained for the use of anonymised radiographic images for publication. Permission to reproduce the manufacturer's biomechanical imagery was obtained from the copyright holder. The manufacturer had no role in study design, data collection, data analysis, interpretation of results, manuscript preparation, or patient selection.

All adult patients undergoing foot arthrodesis during the study period were screened. Inclusion criteria for the main cohort were (1) midfoot or Chopart arthrodesis and (2) use of nitinol compression staples as the principal fixation method, either alone or in combination with adjunct fixation.

Revision arthrodesis procedures, active infection, and Charcot neuroarthropathy were excluded. Patients with diabetes were included provided there was no evidence of Charcot neuroarthropathy. Concomitant procedures were not excluded where a qualifying midfoot or Chopart arthrodesis formed part of the index procedure.

Tarsometatarsal and/or naviculocuneiform arthrodeses were classified as midfoot. Talonavicular and/or calcaneocuboid arthrodeses were classified as Chopart. Procedures involving the talonavicular joint plus adjacent midfoot joints were classified as Chopart constructs due to the biomechanical dominance of the talonavicular joint.

Fixation constructs were categorised as staple-only or hybrid (nitinol compression staples combined with compression screws and/or plates). The use of adjunct fixation was at the discretion of the treating surgeon based on the intraoperative assessment of construct stability. Construct configuration, number of staples, staple geometry and orientation, and the use of adjunct fixation were recorded for each procedure.

Electronic medical records and imaging were reviewed for patient demographics, comorbidities, joints fused, fixation constructs, number and configuration of staples, adjunct fixation, bone graft use, postoperative protocols, radiographic union, complications, and re-operations.

Radiographic union was defined as ≥50% trabecular bridging across the arthrodesis site on two orthogonal radiographs or confirmation on computed tomography. Computed tomography was not performed routinely and was obtained selectively where radiographic union was uncertain or clinical concerns regarding non-union existed. For multi-joint arthrodeses, union was assessed across the overall fusion construct and recorded on a per-procedure basis. Time to union was calculated from the date of surgery to the first documented evidence of union. Complications included wound complications, delayed union, non-union, symptomatic metalwork failure, and re-operation.

Postoperatively, patients were immobilised in a cast or walking boot and remained non-weight-bearing for approximately six weeks. Progressive weight-bearing was then permitted following clinical and radiographic assessment at the discretion of the treating surgeon.

Given the descriptive nature of the study, outcomes were summarised using descriptive statistics only. Continuous variables are presented as means or medians with interquartile ranges and categorical variables as frequencies and percentages.

## Results

Twenty-five midfoot and Chopart arthrodeses met the inclusion criteria. Fifteen procedures involved midfoot joints, and 10 involved Chopart joints. A total of 58 joints were fused, with a median of two joints per procedure. Patient demographics and comorbidities are summarised in Table 1. The median follow-up was 18 months (range: 6-24 months).

**Table 1:** Patient demographics and comorbidities SD: standard deviation

Variable	Value
Number of procedures	25
Mean age years (range)	58.5 (32-68)
Mean BMI, kg/m² (SD)	31.4 (5.3)
Diabetes mellitus, n (%)	3 (12%)
Current smoker, n (%)	5 (20%)
Rheumatoid arthritis, n (%)	2 (8%)
Peripheral vascular disease, n (%)	1 (4%)

Staple-only constructs were most common, with hybrid constructs (staples combined with compression screws and/or plates) used selectively, predominantly in Chopart arthrodesis. Between two and five staples were used per procedure, frequently arranged to provide multi-planar compression across multi-joint fusion masses. Operative characteristics, including anatomical distribution and fixation constructs, are shown in Table 2.

**Table 2:** Operative characteristics and fixation constructs IQR: interquartile range *Hybrid constructs were staples combined with screws and/or plates

Variable	Value
Total joints fused	58
Median joints fused per procedure (IQR)	2 (1-3)
Anatomical region, n (%)	
Midfoot (tarsometatarsal/naviculocuneiform)	15 (60%)
Chopart (talonavicular/calcaneocuboid)	10 (40%)
Fixation construct, n (%)	
Staple-only	20 (80%)
Hybrid constructs*	5 (20%)
Staples per procedure, range	2-5
Bone graft used, n (%)	6 (24%)

Overall union was achieved in 22 of 25 midfoot/Chopart procedures (88%). In the midfoot cohort, 14 of 15 procedures united (93.3%). From the selected Chopart group, eight of 10 procedures united (80%). The median time to union among united procedures was 62 days (IQR: 47-80). Three non-unions occurred: two involving Chopart constructs (one hybrid construct and one staple-only construct) and one involving a midfoot fusion using a staple-only construct.

Three complications were recorded (12%): one wound complication requiring operative debridement, one non-union associated with symptomatic metalwork failure, and one case of persistent postoperative pain managed non-operatively. One case of staple fatigue failure occurred at the bridge-leg interface. Two procedures required further surgery. Radiographic outcomes, time to union, and complications are detailed in Table 3.

**Table 3:** Radiographic outcomes and complications IQR: interquartile range *Calculated among procedures achieving union

Outcome	Value
Radiographic union, n (%)	22 (88%)
Non-union, n (%)	3 (12%)
Median time to union, days (IQR)*	62 (47-80)
Union by region, n/N (%)	
Midfoot	14/15 (93.3%)
Chopart	8/10 (80%)
Any complication, n (%)	3 (12%)
Broken metalwork, n (%)	1 (4%)
Re-operation required, n (%)	2 (8%)

## Discussion

This study demonstrates that nitinol compression staples provide effective fixation for midfoot arthrodesis and acceptable outcomes in Chopart arthrodesis in routine clinical practice. The overall union rate of 88%, with a higher union rate observed in midfoot procedures compared with Chopart procedures, reflects a pattern of region-specific variation reported in recent flagship studies [4].

Midfoot arthrodesis has demonstrated favourable union rates across a range of fixation strategies, including screw, plate, and hybrid constructs, with reported union rates typically ranging from 85% to 95%. Outcomes are influenced by factors including anatomical location, smoking status, comorbidities, and construct selection. Previous studies evaluating both traumatic and degenerative midfoot arthrodesis have reported high union rates using conventional fixation methods [6-9]. These findings suggest that successful arthrodesis depends not only on achieving compression across the fusion site but also on providing sufficient rotational stability to resist multi-planar loading during healing. The union rates observed in the present series compare favourably with those reported using more traditional fixation constructs.

Horner et al. reported union in 63 of 72 midfoot joints (87.5%) compared with 18 of 25 Chopart joints (72%), with the majority of non-unions occurring at the talonavicular joint [3]. Similarly, Dock et al. reported a single non-union among 17 tarsometatarsal arthrodeses using nitinol staples, corresponding to a union rate of 94.1% [2]. In the present study, all but one midfoot arthrodesis achieved union, with a greater proportion of non-unions occurring within Chopart constructs, mirroring these previously reported regional patterns.

The Chopart joint complex, comprising the talonavicular and calcaneocuboid joints, is central to midfoot motion and dynamic foot function. As the functional interface between the hindfoot and midfoot, the transverse tarsal joint permits flexibility during weight acceptance and rigidity during propulsion, exposing the region to substantial shear and rotational forces during gait. The talonavicular joint, in particular, acts as a key pivot for medial column motion and is subjected to high multi-planar stresses that may challenge sustained compression across an arthrodesis. In contrast to the relatively constrained tarsometatarsal joints, this inherent mobility and force transmission may predispose Chopart arthrodesis to non-union. In addition, the talonavicular joint has a relatively limited central blood supply and complex ligamentous attachments, factors that have been associated with delayed healing and higher non-union rates [10,11]. These biomechanical and anatomical characteristics provide a plausible explanation for the lower union rates observed in Chopart arthrodesis compared with midfoot fusion in both the present study and the existing literature.

Previous studies have demonstrated lower union rates following talonavicular and Chopart arthrodesis compared with more constrained tarsometatarsal joints [11,12]. This likely reflects the substantial rotational and shear forces transmitted across the transverse tarsal joint complex during gait, in addition to the relatively limited vascular supply of the talonavicular joint [10-12]. Consequently, adjunct fixation strategies including compression screws, dorsal plating, and hybrid constructs have been advocated in higher-risk Chopart arthrodesis to improve construct stability during healing [13,14].

Construct design appears to be an important determinant of success. A variety of fixation strategies have been described for midfoot and Chopart arthrodeses, including screw fixation, dorsal or plantar plating, compression staples, and hybrid constructs. Biomechanical studies have demonstrated that resistance to rotational and cyclic loading is particularly important in anatomically mobile regions such as the talonavicular joint [11,15]. Consequently, multi-planar construct stability and careful fixation planning are important in Chopart arthrodesis [12,15]. Continuous compression implants such as nitinol staples may offer the additional advantage of sustained dynamic compression across the fusion interface during early healing [4,13,16].

In this series, the majority of arthrodeses were stabilised using a single commercially available nitinol compression staple system (Synthes Elite, DePuy Synthes, Raynham, Massachusetts, United States), reflecting implant availability and routine clinical practice at our centre. Nitinol staples function through a shape-memory alloy mechanism, delivering sustained dynamic compression across the fusion site as the implant returns to its pre-set configuration at body temperature. This continuous compressive force differs from static fixation methods and may enhance fusion biology by maintaining inter-fragmentary compression throughout the early postoperative period [4]. 

**Figure 1:**
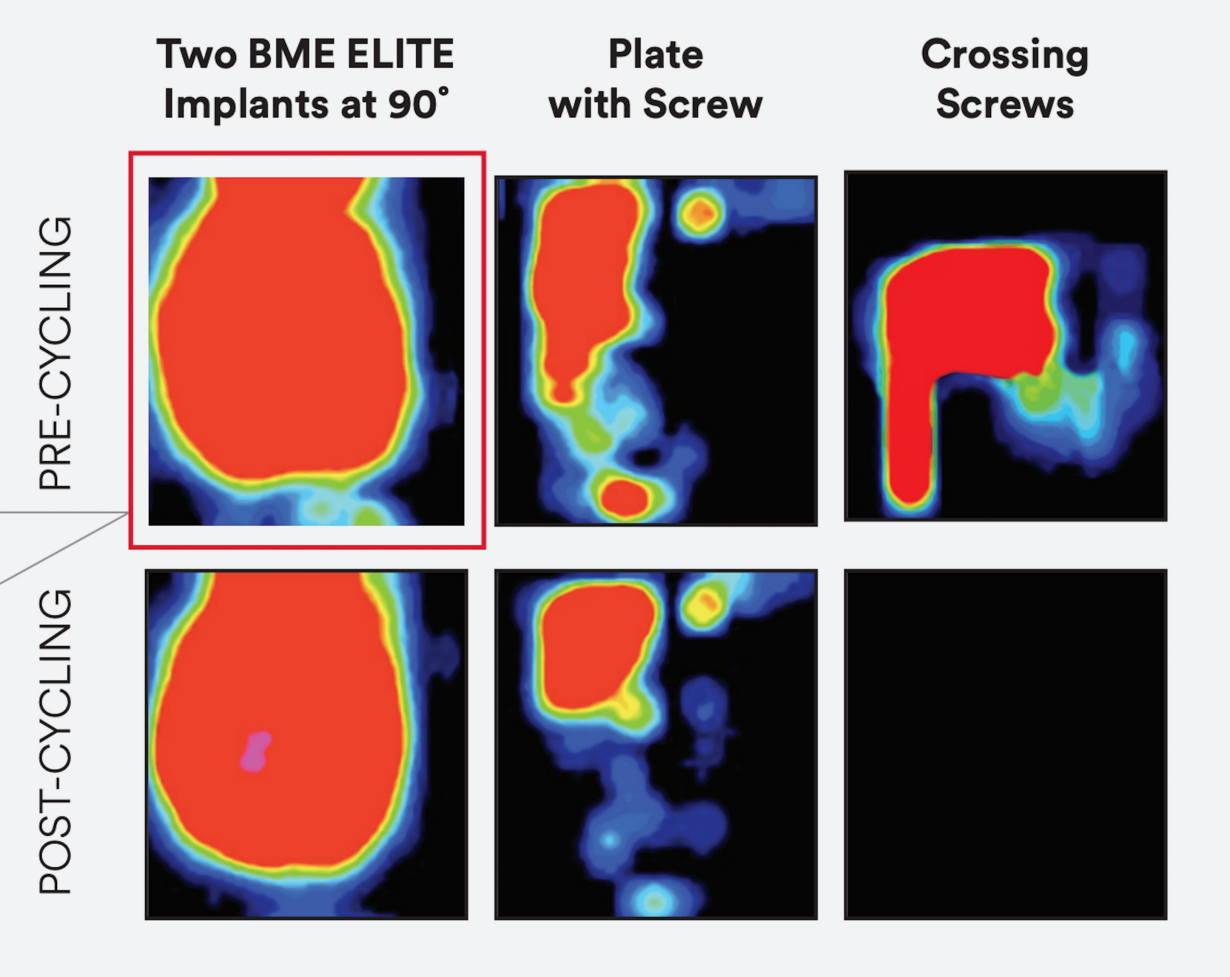
Compression force distribution with nitinol compression staples Representative heat-map images from biomechanical testing demonstrate inter-fragmentary compression across an arthrodesis interface before and after cyclic loading with two orthogonally placed nitinol staples. Warmer colours indicate higher compressive force. Image reproduced from the Elite Nitinol Compression Staples brochure with permission from DePuy Synthes (Raynham, Massachusetts, United States)

The spatial distribution of inter-fragmentary compression generated by continuous compression staples is illustrated in Figure 1, demonstrating the broad surface area over which compressive forces are maintained across the fusion interface.

In this cohort, multiple staples were commonly used, often combining different staple geometries and orientations. Biomechanical rationale suggests that increasing staple number and employing orthogonal rather than purely parallel staple orientation may improve resistance to shear and torsional forces, particularly in Chopart constructs [4]. Four-leg straight staples provide increased surface area contact and improved resistance to rotational forces compared with conventional two-leg staples, while Y-shaped staples are designed to distribute compressive forces across divergent vectors, potentially enhancing stability across anatomically complex or curved joint surfaces [3]. In contrast, traditional two-leg staples provide focused compression but may be more susceptible to torsional loading when used in isolation, particularly in high-stress regions such as the talonavicular joint [8]. Although this study was not powered to formally evaluate the influence of staple number, shape, or orientation, the clustering of non-unions within Chopart constructs supports careful construct planning in this region.

Hybrid constructs incorporating compression screws or plates were used selectively, reflecting real-world practice. While all but one hybrid construct achieved union, it is notable that one non-union occurred in a construct combining nitinol staples with plate fixation. This finding highlights that adjunct fixation does not necessarily mitigate the underlying biomechanical challenges of Chopart arthrodesis and underscores the importance of construct strategy rather than fixation quantity alone.

Mechanical failure of nitinol staples was uncommon in this series. In the single case of staple failure observed, fracture occurred at the bridge-leg interface, consistent with regions of peak stress concentration within the implant. This failure pattern is characteristic of cyclic fatigue rather than acute overload and may arise in the setting of delayed union or persistent motion across the arthrodesis site [13]. Retained staple legs are frequently asymptomatic and do not require removal unless associated with pain or non-union [4].

Nitinol compression staples offer a low-profile construct that may be advantageous in anatomically constrained regions of the foot. In this series, no clinically significant hardware prominence or implant migration requiring revision was observed, supporting the practical appeal of staple fixation in routine practice.

**Figure 2:**
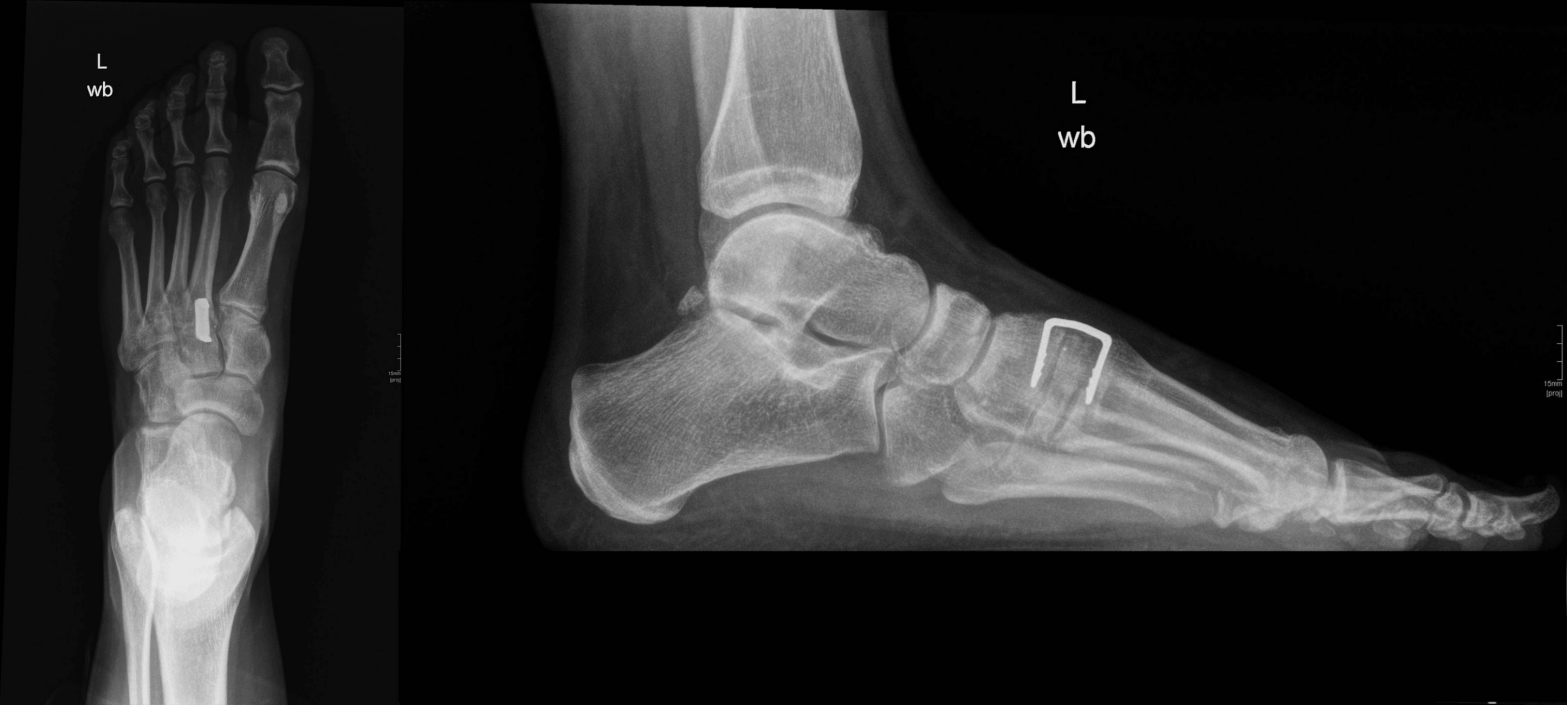
Postoperative weight-bearing radiographs of midfoot arthrodesis using nitinol compression staples, demonstrating maintained alignment and low-profile fixation

The low-profile nature of staple fixation is illustrated in Figure 2, demonstrating maintained alignment and minimal implant prominence on postoperative weight-bearing radiographs.

Complications and re-operations in the present series were infrequent and occurred predominantly in non-midfoot cases. The majority of non-unions and complications were associated with Chopart constructs, further supporting the concept that arthrodesis in this region carries a higher risk profile than midfoot fusion. Overall complication and re-operation rates were comparable with those reported in the literature [4].

The overall non-union and complication rates observed in the present series were broadly comparable with those reported in the wider midfoot and hindfoot arthrodesis literature using conventional fixation methods. Reported non-union rates following midfoot arthrodesis generally range from 5% to 15%, depending on anatomical location, fixation strategy, patient factors, and underlying pathology [7,9,14]. In this context, the present findings suggest that nitinol compression staples can achieve union rates comparable with traditional fixation constructs while offering the potential advantages of lower-profile implants and sustained dynamic compression across the arthrodesis site.

Outcomes in the present study are reported on a per-procedure basis, whereas some comparator studies, including Horner et al., report outcomes per joint. This methodological difference can influence reported percentages, particularly in multi-joint arthrodeses, and should be considered when making direct numerical comparisons [3]. Other limitations include the retrospective design, single-centre setting, modest sample size, lack of a control group, and absence of standardised patient-reported outcome measures. The sample size also limits the ability to perform adequately powered subgroup analyses, particularly when comparing midfoot and Chopart constructs. As with all retrospective studies, selection bias cannot be excluded.

## Conclusions

Nitinol compression staple fixation was associated with high union rates in midfoot arthrodesis and acceptable outcomes in Chopart arthrodesis in routine clinical practice. Outcomes were more predictable in the midfoot than in the Chopart region, consistent with the greater biomechanical demands reported in the existing literature. Careful construct planning and selective adjunct fixation may be particularly important in higher-risk Chopart arthrodesis.
